# Thiamethoxam Application Improves Yield and Drought Resistance of Potatoes (*Solanum tuberosum* L.)

**DOI:** 10.3390/plants13040477

**Published:** 2024-02-07

**Authors:** Hailong Qiu, Chao Sun, Richard Dormatey, Jiangping Bai, Zhenzhen Bi, Yuhui Liu, Zhen Liu, Jingui Wei, Shoufa Mao, Panfeng Yao

**Affiliations:** 1 State Key Laboratory of Aridland Crop Science/College of Agronomy, Gansu Agricultural University, Lanzhou 730070, China; 17393164380@163.com (H.Q.); sunc@gsau.edu.cn (C.S.); rmddormatey@gmail.com (R.D.); baijp@gsau.edu.cn (J.B.); bizz@gsau.edu.cn (Z.B.); lyhui@gsau.edu.cn (Y.L.); liuzhen@gsau.edu.cn (Z.L.); weijg17797691770@163.com (J.W.); maoshoufa@163.com (S.M.); 2 CSIR-Crops Research Institute, P.O. Box 3785, Kumasi 00233, Ghana

**Keywords:** potato, thiamethoxam, drought resistance, malondialdehyde, yield

## Abstract

(1) Background: Potato is the most important tuber crop in the world that can contribute to food security. However, the crop has been shown to be sensitive to drought and its yields decline significantly during successive periods of stress. Drought triggers a number of responses in potato, ranging from physiological changes to fluctuations in growth rates and yields. In light of global climate change, it is important to understand the effects of thiamethoxam on potato growth and yield under drought conditions. (2) Methods: The objective was to evaluate the impact of thiamethoxam on improving drought resistance and yield of potato under drought conditions. The drought-tolerant and sensitive-genotypes Qingshu No. 9 and Atlantic were used for a two–year pot experiment. Potato seeds were coated with 70% thiamethoxam before sowing (treatment group (T)), with a control group without treatment (NT). Two experimental treatments were applied: normal irrigation (ND) and drought stress (D). (3) Results: The results showed that root length, plant yield, chlorophyll content and superoxide dismutase (SOD) activity significantly increased under both genotypes, while malondialdehyde (MDA) and proline (Pro) content were reduced under thiamethoxam under drought stress. The best indicators were obtained in the comprehensive evaluation for the T–D treatment, suggesting that the application of thiamethoxam under drought stress was more effective than normal irrigation. (4) Conclusions: Our results suggest that the application of thiamethoxam improves potato growth, thereby increasing drought tolerance and potato yield. However, thiamethoxam is a neonicotinoid pesticide, and the limitation of this study is that it did not explore the ecological effects of thiamethoxam, which need to be systematically studied in the future. Moreover, considering the potential risks of thiamethoxam to the environment, specific agronomic measures to effectively degrade thiamethoxam residue should be taken when it is applied in agricultural production.

## 1. Introduction

Potato (*Solanum tuberosum* L.) is the fourth-largest food crop in the world [1]. Its tubers are highly nutritious and provide more proteins, carbohydrates, vitamins, and minerals than other food crops [2]. Potatoes play an important role in ensuring national food security and are widely used in industry, livestock and food processing [3,4]. In 2015, China introduced a strategy to develop potato as a staple food, which has greatly promoted the development of China’s potato industry [5]. However, Chinese potato growing areas are mainly located in arid and semi-arid hilly mountainous regions, and one of the main problems of agricultural production in these areas is the changeable harsh climate and severe water shortage [6]. Long-term or seasonal drought stress can seriously affect potato plant growth, tuber yield, and profitability [7,8]. Therefore, drought is an important factor limiting potato production [9,10], which requires urgent study in terms of how to improve drought resistance and yield enhancement of potatoes.

Some indicators of potato drought resistance evaluation analysis and summary show that the current methods of potato drought resistance determination are mainly determined by the change in morphological, physiological and biochemical indicators of potato under drought stress [11,12]. Drought stress inhibits the increase in potato plant height and stem thickness, and shows significant damage and changes in root growth and phenotype, as well as affecting the relative chlorophyll content of potato, ultimately reducing yield [13]. Drought stress also affects physiological and biochemical processes in potato, with changes in photosynthetic rate, MDA, Pro and SOD content [14]. A decrease in relative chlorophyll content leads to a decrease in photosynthetic rate [15]. Drought stress leads to the accumulation of reactive oxygen species in potato, resulting in the production of large amounts of MDA, causing cytoplasmic membrane damage and inhibiting physiological and biochemical processes, while Pro prevents osmotic stress from harming the plant and protects the normal function of polysaccharides [14,16]. In order to cope with excessive reactive oxygen species in the body and reduce the damage of adversity, potatoes will produce a large amount of SOD through the antioxidant enzyme system to protect the plants [17]. Therefore, the above indicators can be used as the basis for identifying the drought resistance of potatoes.

One way to improve the drought resistance of potatoes is to cultivate drought-resistant varieties. Another way is to use drought-resistant agricultural measures. However, the time required for the development of drought-resistant varieties is generally long, and their performance varies in different areas [18]. Therefore, it is a more reliable choice to adopt appropriate agricultural measures and explore the drought resistance of some pesticides, in order to adjust the potato’s response to drought [19]. Ruisheng is a class of seed treatments manufactured by Syngenta containing 70% thiamethoxam, a highly effective and low-toxicity insecticide of the second generation of neonicotinoids [20]. This active ingredient has become an important component of insect control programs worldwide due to its broad-spectrum insecticidal properties, low application rate, long duration of action, and excellent uptake and utilization by plants [21]. Previous studies have shown that thiamethoxam can activate plant proteins related to plant stress resistance [22], improve the germination rate of spring wheat and other crops, promote the development of the root system, increase the dry weight of spikes and the number of fertile tillers [23,24], and effectively increase crop yield [25]. These benefits are not achieved by other insecticides. Thiamethoxam has high water solubility, so it works well under various environmental conditions, and significantly improves crop yield and drought resistance [26,27]. However, the specific effect and mechanism of thiamethoxam on drought resistance or tolerance and yield enhancement in potato are still unclear and need to be further studied.

In this study, seed potatoes were planted after thiamethoxam coating, and the effects of drought stress on growth status, photosynthetic effect, physiological and biochemical indices, and final yield of potatoes at different time points were analyzed. In addition, principal component analysis and the membership function method were employed to comprehensively evaluate the effects of thiamethoxam as a seed-coating agent on potato growth and development. This provided a basis for analyzing the drought resistance and yield enhancement mechanism of thiamethoxam.

## 2. Results

### 2.1. Effect of Thiamethoxam on Plant Height of Potatoes in Different Treatments

**Figure 1:**
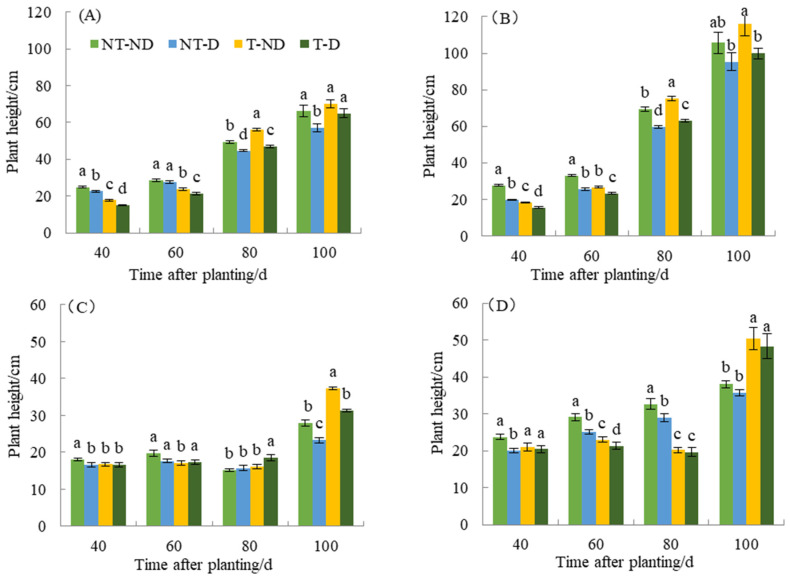
Differences in plant height (cm) of two potato genotypes under four different treatments in two growing seasons. (**A**) Atlantic, planted in 2018; (**B**) Qingshu No. 9, planted in 2018; (**C**) Atlantic in 2019; and (**D**) Qingshu No. 9 in 2019. Values represent the mean of 3 replicates ± standard deviation (SD). Bars with different lowercase letters indicate significant differences by Duncan multiple range test (*p* ≤ 0.05).

There was a significant difference between the treatments with the two potato genotypes in terms of plant height in the 2018 and 2019 cropping seasons (Figure 1A–D). Thiamethoxam-treated plants (T–ND and T–D) had a reducing effect on both Atlantic and Qingshu No. 9 potato genotypes in the first growth phase. Thus, 40 and 60 days after planting, thiamethoxam-treated plants (T–ND and T–D) showed significant reductions in plant height of 28.61% and 40.89% and 19.77% and 22.17% compared to the corresponding control plants in the Atlantic genotype. A similar trend was observed in the Qingshu No. 9 genotype. The height of thiamethoxam-treated plants (T–ND and T–D) significantly decreased by 34.32% and 21.26% and 19.20% and 8.76% 40 and 60 days after planting, respectively. In addition, both genotypes showed significant plant height among the four treatments 80 days after planting. Treatment T–ND had the highest plant height, followed by NT–ND, T–D, and NT–D with the lowest plant height. However, at 100 days after planting, there was no significant difference among treatments T–ND, NT–ND, and T–D, except that treatment NT–D had the lowest plant height in genotype Atlantic. In Qingshu No. 9, treatment T–ND had the highest plant height and there was no significant difference between the other treatments 100 days after planting in the 2018 growing season. In the 2019 growing season, the results show that the control plants had the highest plant height at 40 and 60 days compared to the other treatments. However, plants treated with thiamethoxam in treatment T–D had the highest plant height 80 days after planting compared to the other treatments. At 100 days after planting, T–ND had the highest plant height, followed by T–D and NT–ND, while NT–D had the lowest plant height in the Atlantic genotype. Moreover, at 40, 60 and 100 days after planting, the control plants (NT–ND) had the highest plant height compared to the other treatments (DAP), but 100 days after planting, the thiamethoxam-treated plants (T–ND and T–D) had the highest plant height compared to the control plants of genotype Qingshu No. 9 in 2019.

### 2.2. Effect of Thiamethoxam on Potato Stem Thickness under Different Treatments

**Figure 2:**
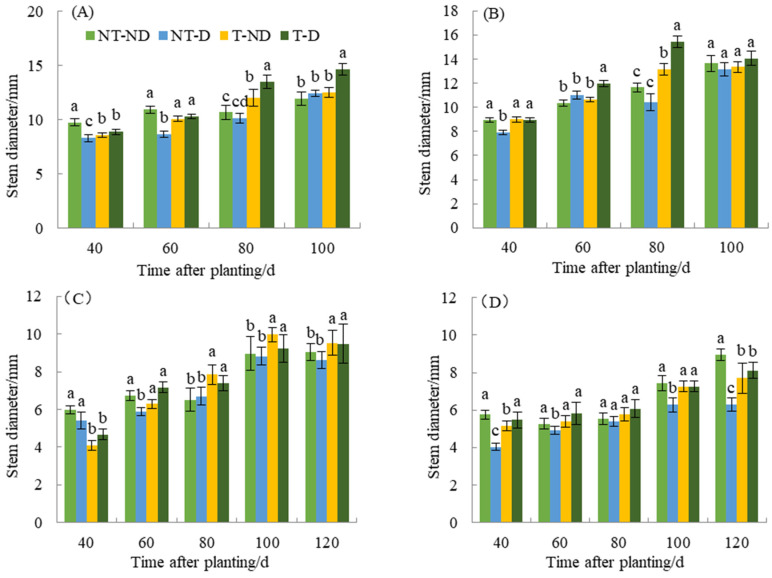
Differences in stem diameter (mm) of two potato genotypes under four different treatments in two growing seasons. (**A**) Atlantic, planted in 2018; (**B**) Qingshu No. 9, planted in 2018; (**C**) Atlantic in 2019; and (**D**) Qingshu No. 9 in 2019. Values represent the mean of 3 replicates ± standard deviation (SD). Bars with different lowercase letters indicate significant differences by Duncan multiple range test (*p* ≤ 0.05).

Stem thickness resulting under different treatments of the two genotypes in the 2018 and 2019 planting seasons showed significant differences (Figure 2). At 40 DAP, the control plants had the highest stem thickness, while the (NT–D and T–D) had the lowest. In addition, treatment T–D and control had the highest stem thickness, while NT–D had the lowest at 60 DAP. At 80 and 100 DAP, T–D recorded the highest plant height, while the control and NT–D had the lowest stem thickness. In genotype Qingshu No. 9, the control and T–D had the highest stem thickness, while NT–D had the lowest at 40, 60 and 80 DAP. At 100 DAP, NT–D had the least stem thickness, while there was no significant difference in stem thickness among the other treatments. At 40 DAP in 2019, control plants and NT–D had the greatest stem thickness, while T–D and T–ND had the least. At 60 DAP, the control and T–D plants had the highest value, while the NT–D plant had the lowest value of stem thickness. At 80, 100 and 120 DAP, the thiamethoxam-treated plants (T–D and T–ND) had the highest stem thickness value, while the untreated plants had the lowest value for the Atlantic genotype. Moreover, the results of Qingshu No. 9 were similar to those of Atlantic genotype in the same planting season. The control and T–D plants showed higher values of stem thickness at 40 DAP compared to NT–D treatment. Stem thickness at 60, 80 and 100 DAP, treatments T–D and T–ND recorded the highest values, while the NT–D had the lowest value of stem thickness. During the two years of different growth periods, the stem thickness of T–D of Qingshu No. 9 was elevated by an average of 20.78% compared with that of NT–D, while that of Atlantic Ocean was elevated by an average of 13.01%, with the increase in stem thickness of Qingshu No. 9 being more significant.

### 2.3. Effect of Thiamethoxam on Root Length of Potato under Different Treatments

**Figure 3:**
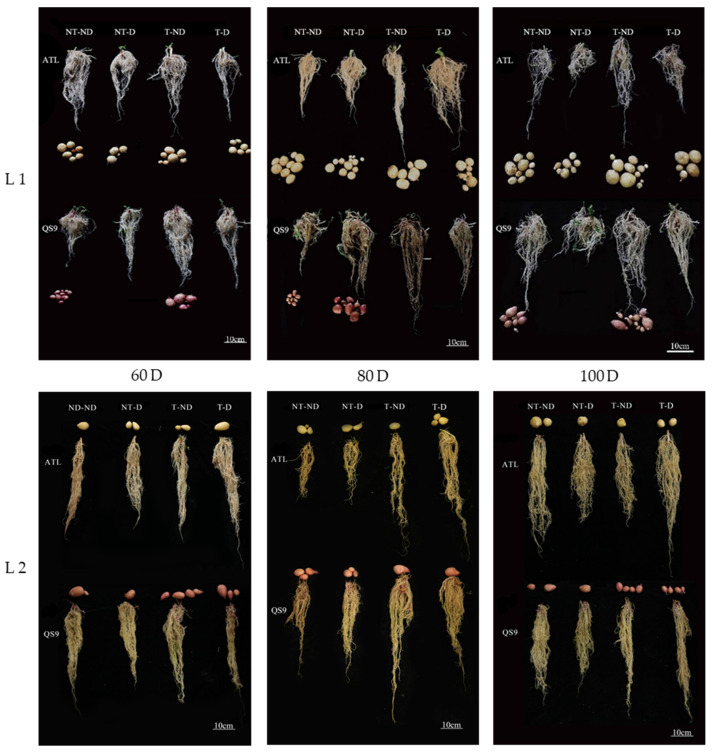
Differences in root length (cm) of two potato genotypes under four different treatments in two growing seasons. L1: Root length in 2018; L2: root length in 2019; 60 D, 80 D and 100 Dr respectively, refer to potato growth periods of 60 days, 80 days and 100 days.

Because deep soils are less affected by external changes and the soil environment is relatively stable, changes in the deep root system are more informative for studying drought resistance in plants. Images of the root systems of two potato genotypes under different treatments in both growing seasons showed significant differences in root length between treatments (Figure 3). In 2018, the results showed that root length was significantly higher under treatment T–ND, followed by treatment T–D, and the lowest root length was observed for the NT–D in genotype Atlantic at 60, 80 and 100 DAP. In the Qingshu No. 9 genotype, T–ND had the highest root length, followed by T–D and the control plants, with the least root length at 60, 80 and 100 DAP. Moreover, plants under treatment T–ND showed the highest root length, followed by treatment T–D at 60, 80 DAP. In 100 DAP, T–D and NT group had the highest root length, followed by T–ND and NT–ND, and NT–D had the lowest root length in the Atlantic genotype in the 2019 growing season. In addition, the highest root length was obtained in treatment T–ND, followed by treatment T–D, while the control plants had the lowest value at 60 and 80 DAP in Qingshu No. 9. Treatment T–ND exhibited the highest root length, followed by T–D and NT group exhibited the lowest root length among the treatments. The increase in root length of thiamethoxam-treated plants in the treatments (T–D and T–ND) indicates that thiamethoxam can effectively increase the root length of potato to absorb deep soil water when potato plants are exposed to drought stress. This increases the drought resistance of potato. With sufficient soil moisture, thiamethoxam also has a positive effect on potato root development.

### 2.4. Effect of Thiamethoxam on Relative Chlorophyll Content in Potatoes

**Figure 4:**
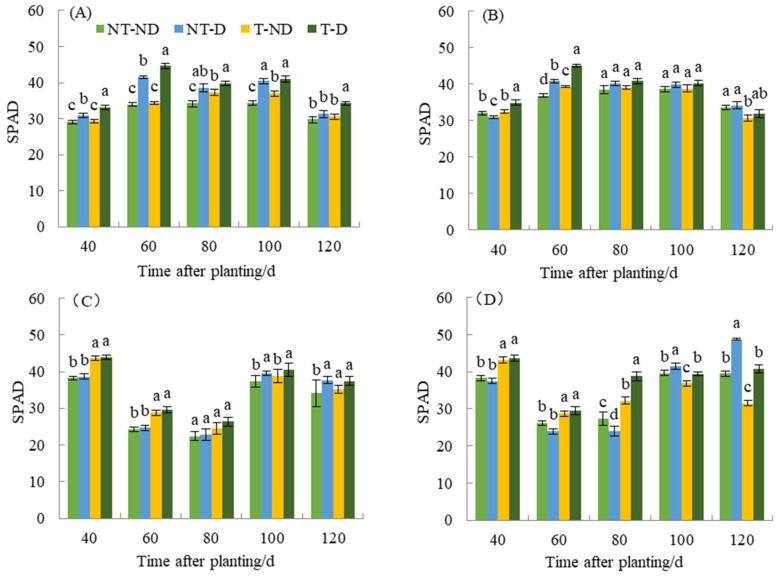
Differences in relative chlorophyll content of two potato genotypes under four different treatments in two growing seasons. (**A**) Atlantic, planted in 2018; (**B**) Qingshu No. 9, planted in 2018; (**C**) Atlantic in 2019; and (**D**) Qingshu No. 9 in 2019. Values represent the mean of 3 replicates ± standard deviation (SD). Bars with different lowercase letters indicate significant differences by Duncan multiple range test (*p* ≤ 0.05).

Chlorophyll content has an important effect on photosynthesis, morphology, and final yield of plants. The relative chlorophyll content of the two genotypes under different treatments in the 2018 and 2019 growing seasons showed significant differences (Figure 4). In 2018, the relative chlorophyll content of plants under the T treatment was higher than that of the NT group throughout the reproductive period of Atlantic, with the relative chlorophyll content of the T–D treatment being 7.20–9.37% higher than that of the NT–D treatment at 40, 60, and 120 DAP. This phenomenon was also observed no. 9. during 40–100 DAP of fertility. In 2019, the relative chlorophyll contents of T–ND and T–D treatments were significantly higher than those of NT group at 40–100 DAP of Atlantic fertility, in which the T–D treatment was significantly higher than NT–D treatment by 13.70% and 19.73% at 40 and 60 DAP. The above phenomenon was also observed at 40, 60, and 80 DAP of the fertility of No. 9, in which the T–D treatment was significantly higher than NT–D treatment by 16.54–61.65%. The SPAD value of T–D of Qingshu No. 9 increased by 9.89% compared with NT–D on average during different growth periods in the two years, while Atlantic increased by an average of 7.92%, and the increase in SPAD values of Qingshu No. 9 was more significant.

### 2.5. Effect of Thiamethoxam on MDA Content in Potatoes

**Figure 5:**
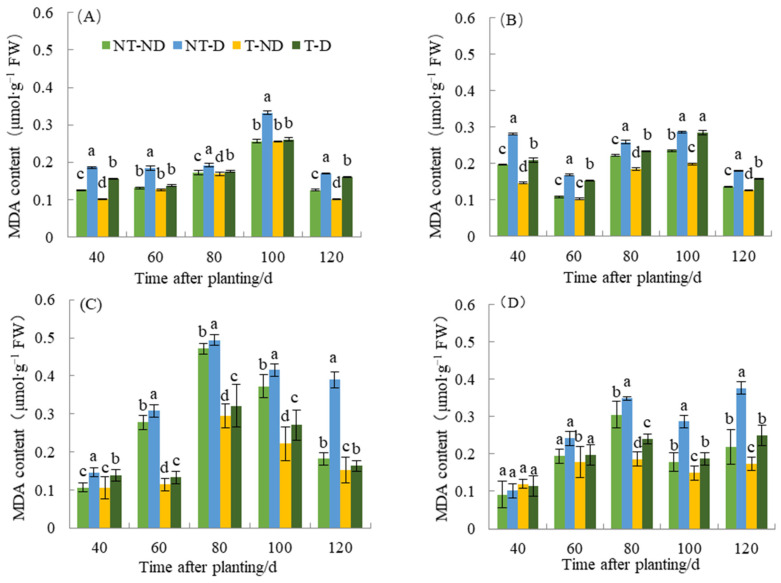
Differences in malondialdehyde content of two potato genotypes under four different treatments in two growing seasons. (**A**) Atlantic, planted in 2018; (**B**) Qingshu No. 9, planted in 2018; (**C**) Atlantic in 2019; and (**D**) Qingshu No. 9 in 2019. Values represent the mean of 3 replicates ± standard deviation (SD). Bars with different lowercase letters indicate significant differences by Duncan multiple range test (*p* ≤ 0.05).

Plants undergo membrane lipid peroxidation under unfavorable conditions, such as drought, and the end product of the membrane lipid peroxidation reaction is mainly MDA. Malonaldehyde is an important factor in membrane system damage and may serve as an indicator of plant resistance to adverse conditions under certain conditions. Stress leads to increased lipid peroxidation of membranes and thus to an increase in MDA content. This led to a cross-linking reaction and denaturation of nucleic acids and proteins and accelerated the aging of plants. The results of MDA content in relation to the four treatments in both genotypes showed significant differences (Figure 5). The results showed that potato plants under NT–D treatments had the highest MDA level compared to the other treatments at 40, 60, 80, 100 and 120 in both cropping seasons in the two genotypes. In both cropping seasons, treatments T–D recorded significantly reduced MAD content values at 40, 60, 80, 100 and 120 DAP, respectively, with respect to the two genotypes. The reduction in MDA content was (5.32–58.02%) in Atlantic, while 0.58–34.70% occurred in Qingshu No. 9. The reduction in MDA content was observed in thiamethoxam-treated plants in the treatments (T–D and T–ND) compared to the control groups (NT–ND and NT–D). During the two years of different fertility periods, the MDA content of T–D was reduced by an average of 26.8% in Atlantic compared to NT–D, whereas it was reduced by 16.3% in Qingshu No. 9, and the MDA content in Atlantic decreased more significant. This observation is probably due to the effect of thiamethoxam, which can effectively reduce the content of MAD in potatoes when they suffer from adversity and reduce the toxicity of MDA in potatoes.

### 2.6. Effect of Thiamethoxam on Potato Pro Content

**Figure 6:**
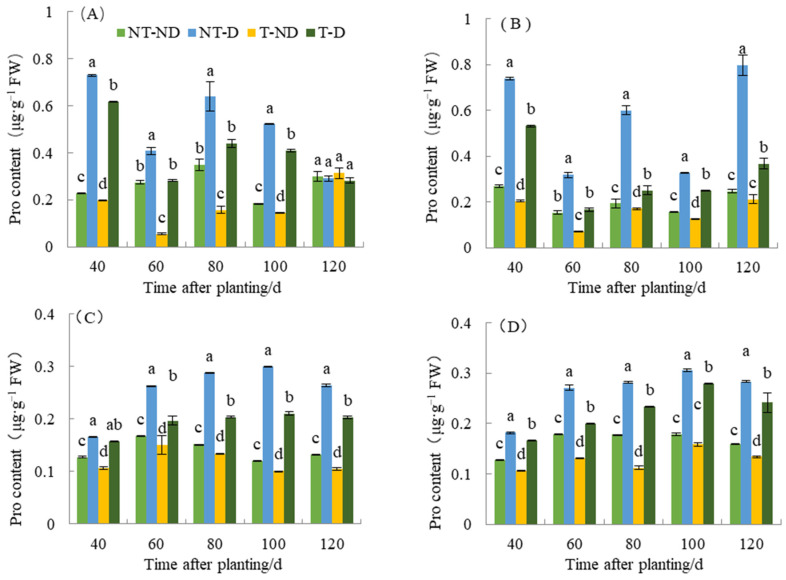
Differences in proline content of two potato genotypes under four different treatments in two growing seasons. (**A**) Atlantic, planted in 2018; (**B**) Qingshu No. 9, planted in 2018; (**C**) Atlantic in 2019; and (**D**) Qingshu No. 9 in 2019. Values represent the mean of 3 replicates ± standard deviation (SD). Bars with different lowercase letters indicate significant differences by Duncan multiple range test (*p* ≤ 0.05).

Proline (Pro) is one of the components of plant proteins and can be widely distributed in free state in the plant system. The accumulation of proline content in plants reflects to some extent the stress resistance status of plants. Plants that are resistant to stresses such as drought stress tend to accumulate more proline. Therefore, determination of proline content can be used as a physiological indicator in breeding drought-resistant plants. As shown in Figure 6, proline content under different treatments of the two genotypes in the 2018 and 2019 growing seasons showed significant differences between treatments. The treatments of the two potato genotypes produced significantly different results with respect to the different time periods. Plants under NT–D showed the highest Pro value, followed by those under T–D at 40, 60, 80 and 100 DAP in both Atlantic and Qingshu No. 9 in both 2018 and 2019 cropping seasons. Potato plants under treatments T–ND had the lowest Pro content values at almost all time periods for both genotypes in the two cropping seasons. In general, the proline content of the two varieties increased significantly under drought stress compared with normal irrigation. However, in both genotypes, thiamethoxam significantly reduced proline content under both drought stress and normal soil moisture conditions. For example, a maximum reduction of 58.12% in proline content was observed in the T–D treatment compared to the NT–D treatment in Qingshu No. 9 in 2018. It is possible that thiamethoxam reduces the potato’s sensitivity to drought and caused this phenomenon such that potato can successfully cope with drought damage by producing less proline content under drought stress. In addition, during different growth periods over two years, the Pro content of T–D of Qingshu No. 9 exhibited an average decrease of 28.66% compared to NT–D, and the Atlantic experienced a reduction of 21.48%. The Pro content of Qingshu No. 9 showed a greater decrease. This phenomenon is side evidence that thiamethoxam can improve drought resistance in potato, but its detailed mechanism needs to be further studied in the future.

### 2.7. Effect of Thiamethoxam on SOD Content in Potatoes

**Figure 7:**
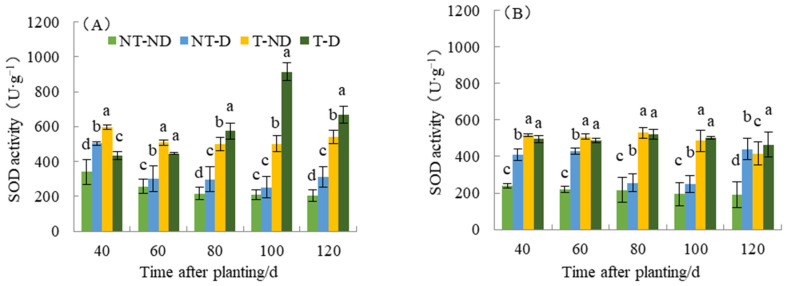
Differences in superoxide dismutase activity of two potato genotypes under four different treatments in two growing seasons. (**A**) Atlantic in 2019; and (**B**) Qingshu No. 9 in 2019. Values represent the mean of 3 replicates ± standard deviation (SD). Bars with different lowercase letters indicate significant differences by Duncan multiple range test (*p* ≤ 0.05).

Superoxide dismutase (SOD) eliminates toxic substances generated in cells. When the activity of the enzyme SOD increases, it means that it is a metabolic regulation of adversity in the plant, since the number of superoxide radicals produced by plants increases when they are exposed to adversity. To resist the damage caused by adversity, the activity of the enzyme SOD increases to remove superoxide radicals and reduce lipid peroxidation of the membrane. Results from SOD show significant differences with respect to the four treatments of the two potato genotypes (see Figure 7). For the Atlantic genotype, treatment T–ND had the highest SOD value, followed by NT–D, with NT–ND (control) having the lowest SOD value at 40 DAP. Potato plants under treatments T–ND and T–D had the highest SOD value, while treatments NT–ND had the lowest SOD values at 60 and 80 DAP, while treatments NT–ND had the significantly lowest value. At 100 and 120 DAP, plants under treatment T–D had the highest SOD value, while the control had the lowest. In genotype Qingshu No. 9, plants under treatment (T–ND and T–D) recorded the highest SOD values at 40, 60, 80 and 100, respectively, while treatment NT–ND had the lowest value. At 120 DAP, treatment T–D recorded the highest SOD value followed by NT–D, and NT–ND had the lowest value. The SOD content of T–D at different fertility periods in Atlantic increased by an average of 100.57% compared with that of NT–D, and increased by 48.69% in Qingshu No. 9. This result shows that thiamethoxam can effectively increase the SOD content of potato, which increases the SOD content of potato under drought conditions and reduces the superoxide radicals generated by adverse weather conditions in plants under such conditions. In particular, the Atlantic Ocean showed the largest increase. This improves the drought resistance of potato under drought stress.

### 2.8. Effect of Thiamethoxam Priming on Potato Yield Per Plant

**Figure 8:**
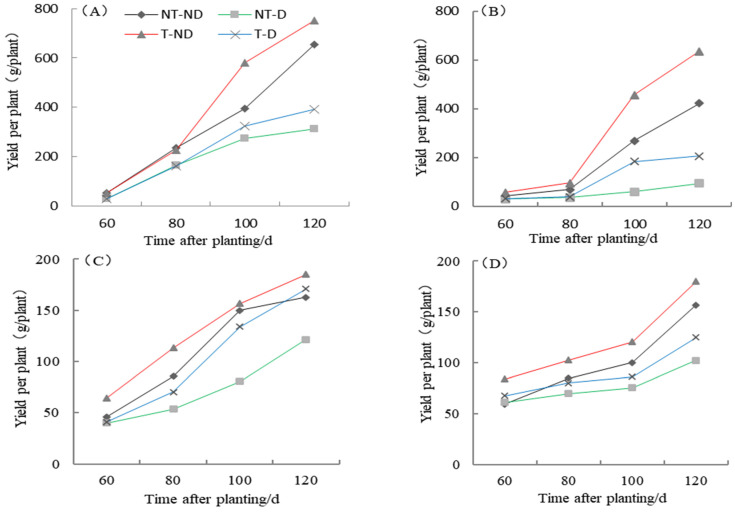
Differences in potato yield per plant of two potato genotypes under four different treatments in two growing seasons. (**A**) Atlantic, planted in 2018; (**B**) Qingshu No. 9, planted in 2018; (**C**) Atlantic in 2019; and (**D**) Qingshu No. 9 in 2019. Values represent the mean of 3 replicates ± standard deviation (SD). Lines differ significantly by Duncan multiple range test (*p* ≤ 0.05).

The yield of the two potato genotypes Atlantic and Qingshu No. 9 under four treatments in the 2018 and 2019 growing seasons showed significantly different results (Figure 8). The results showed that treatment T–ND gave the highest yield followed by control, treatment T–D gave the third highest yield and NT–D gave the lowest yield in both genotypes in the 2018 cropping season. In the 2019 growing season, similar results were obtained for both genotypes. In general, the yields of both genotypes were higher than in 2018. The yield of both genotypes increased the longer the data were collected, from a lower to a higher period: 60–120 DAP. Potato plants under treatment T–ND had the highest yield per plant in all periods, and control plants followed in periods 60 to 100 DAP. Potato plants under T–D treatment recorded the third-highest value at 60 to 100 DAP, but the second highest at 120 DAP in the Atlantic genotype. In genotype Qingshu No. 9, plants under NT–D recorded the highest yield followed by control, plants under T–D treatment recorded the third highest, while NT–D recorded the lowest yield in all periods. From the two-year harvest (120 DAP) yield per plant, it was found that the two-year average increase in T–D over NT–D for Qingshu No. 9 was 70.1% and Atlantic was 33.01%. From these results, it can be concluded that thiamethoxam can increase potato yield, both under suitable soil moisture and drought stress, as thiamethoxam significantly improved potato yield and greatly enhanced the yield of Qingshu No. 9 (drought-tolerant genotype).

### 2.9. Comprehensive Factor Analysis of Potato Drought Resistance Indexes under Different Treatments

Principal component analysis of the potato drought–resistance index based on different treatments revealed two principal components with trait value > 1, as shown in Table 1. Their contribution was 59.71% and 27.97%, respectively, with a cumulative contribution rate of 87.68%. Therefore, the two values of principal component matrix and the contribution rate of each feature value are extracted to calculate the weighting value of each index value, and then the membership function value of each index is calculated for different treatments. According to the membership function value and the weight value of each index, the comprehensive index of each treatment measure is obtained by synthesizing the addition and multiplication rule, and the ranking is based on the size. As shown in Table 2, the total indices of various indicators of drought resistance of potatoes under different treatments were 0.396, 0.309, 0.593, and 0.641, corresponding to NT–ND, NT–D, T–ND, and T–D, respectively. The comprehensive order of treatments in accordance with the resistance level is T–D > T–ND > NT–ND > NT–D. This result shows that the comprehensive factors and indices of potato treatment T–D were the best.

## 3. Discussion

Drought is a common environmental stress that has serious consequences for crop growth, development, and geographic dispersal, as well as for agriculture and food supply. The effects of drought on agricultural productivity can be very detrimental, and in severe cases result in large economic losses. Previous research on drought tolerance in potatoes has shown good results in improving drought resistance in potatoes to achieve higher yields. Other methods include breeding new drought-resistant varieties or using ridging to increase yield and income. In the present study, two potato genotypes with different tolerance traits to drought stress were used to conduct the physiological studies with thiamethoxam to improve drought resistance in potato. The important physiological indices used in this study included plant height, MDA, proline, chlorophyll content, SOD, yield and other root morphological indices of the plant. The experiment was conducted in a two-year pot trial in the 2018 and 2019 growing seasons.

One of the most important guarantors of high crop yields is good plant morphology [28]. These plant morphological characteristics include plant height, stem thickness, root length, and other different postures that occur in plants. These properties help the plant to make the best use of external environmental conditions to ensure normal physiological metabolism and increase biomass accumulation under drought stress conditions [29]. In our study on the effects of thiamethoxam on potato phenotype, thiamethoxam was found to have an inhibitory effect on potato plant height 40 to 60 days after planting. However, after 60 days of growth, the effect of thiamethoxam was found to have a positive effect on plants, as it promoted plant growth. In potato plants treated with thiamethoxam, there was a significant change in growth and development in terms of plant morphology, which was reflected in good yields of potato tubers. Both photosynthetic area and photosynthetic rate increased to allow the accumulation of photosynthetic substances. Stem thickness and rooting depth of plants can be used as important indicators for studying the growth and tolerance status of plants [30]. In the present study, the stem thickness and root length of thiamethoxam-treated plants were significantly greater than the stem thickness and root length of untreated potato plants. This was observed to a greater extent in plants exposed to drought, especially in the late growth period of potato (about 60–100 days). During this period, the plants grew vigorously and the tubers expanded rapidly, which favored the development of stem thickness and root length. The positive effects of thiamethoxam on potato plants during this drought period improved the potato material’s ability to transport and absorb water from depth, improving the potato’s drought resistance. Through research, it was also found that under drought stress, thimerosal promoted root length more significantly in Atlantic (drought-sensitive genotype), but increased stem thickness more in Qingshu No. 9 (drought-tolerant genotype).

According to Huang et al. [31], potato seedlings treated with thiamethoxam showed that the compound was not only effective in controlling below-ground pests, but also significantly increased the chlorophyll content of leaves and improved the seedlings’ resistance to drought stress. In the present studies, the results for both 2018 and 2019 showed a significant increase in chlorophyll content of thiamethoxam-treated potato leaves under drought compared to the responding control plants and the rest of the treatments for all days (40, 60, 80, 100, and 120 days). These results are in agreement with the findings of Lauxen et al. [32] who reported significant increases in chlorophyll content of leaves of *Gossypium* spp. seedlings treated with thiamethoxam under water stress. However, the results of several studies showed that some degree of drought or salt stress can lead to an increase in SPAD value in the plant [33,34]. Rolando et al. [15] postulated that the increase in SPAD values of plants under stress could be due to the decrease in “expansion growth” of leaves by following the measurement of SPAD value of the plant under stress. The results of our experiments also showed that potato plants treated with thiamethoxam under drought conditions had a marginal increase in SPAD levels compared to control plants. Moreover, thiamethoxam more significantly enhanced the chlorophyll content of Qingshu No. 9 (drought-tolerant genotype).

Malondialdehyde (MDA) content is a product of lipid peroxidation that is often used as a measure of oxidative stress in drought stress [35,36]. High accumulation of MDA is often associated with sensitivity to water deficit and stress [37]. The decreased activity of PS II under drought is related to oxidative stress and cell membrane damage caused by increased lipid peroxidation [38]. Our results showed that higher MDA concentration in drought-stressed plants without thiamethoxam treatment was associated with the effects of drought stress on plants. The significant increase in MDA content with progressive drought stress in the two potato genotypes suggests that drought stress causes oxidative damage at all levels in both cultivars, with damage being most severe 80 to 100 days after planting. Our results showed that higher MDA concentration in drought-stressed plants without thiamethoxam treatment was associated with the effects of drought stress on plants. The significant increase in MDA content with progressive drought stress in the two potato genotypes suggests that drought stress causes oxidative damage at all levels in both cultivars, with damage being most severe 80 to 100 days after planting. Previous studies also showed increased MDA content in olive (*Canarium album* (Lour.) Raeusch.) and poplar plants (*Populus* L.) under drought stress [39,40]. The MDA content of thiamethoxam-treated potatoes under drought stress was significantly lower than that under drought stress without thiamethoxam treatment, proving that thiamethoxam can effectively reduce the MDA content of potatoes under drought stress. This result is consistent with the findings of Endres et al. [41], who reported that thiamethoxam significantly increased plant height, root system and dry weight at seedling stage of sugarcane (*Saccharum officinarum* L.) and improved plant photosynthesis under drought stress by reducing the accumulation of MDA content. The experimental results also showed that thiamethoxam was more beneficial in reducing the MDA content of the Atlantic drought-sensitive genotype under drought stress.

Proline is an important organic substance that accumulates and increases in plants under drought conditions [16] and acts as an ROS scavenging molecule [42]. Many previous studies have reported that drought-tolerant plants have higher proline accumulation under drought stress conditions [43,44]. The results from the two seasons for both genotypes confirmed this finding. The proline content of the drought-tolerant potato genotype Qingshu No. 9 showed higher proline content than the drought-sensitive genotype Atlantic, but further observations showed that plants treated with thiamethoxam had significantly lower proline content than untreated control plants. High accumulation of proline in cells under stress conditions protects cells subjected to stress conditions and prevents toxicity from affecting plant cells [45]. Proline also affects osmoregulation, membrane stability, detoxification, pH adjustment of cytosol, and maintaining the structure of enzymes in a cell [44]. However, in this experiment, thiamethoxam was found to be effective in reducing the proline content of potato when subjected to drought stress, and it is hypothesized that thiamethoxam has the potential to reduce the potato’s susceptibility to drought such that it produces less proline content under drought stress and thus successfully copes with the damage sustained in times of drought. Under drought stress, the reduction in Pro content of Qingshu No. 9 (drought-tolerant genotype) by thiamethoxam was more obvious. This explains why thiamethoxam has a positive effect on drought tolerance in potato and could be used as a reliable means of reducing drought stress effects in drought-sensitive genotypes, but its detailed mechanism needs to be further studied in the future.

Antioxidant enzymes play a crucial role in protecting plant cells from stress-induced cellular damage caused by the formation of free radicals, mainly in the form of ROS [46,47]. Therefore, it has been suggested that increasing the activity of antioxidant enzymes under stress may improve plant growth and yield. Our current experiment provided similar results: antioxidant enzyme (SOD) activity increased in all tolerance levels of genotype (40–120) days compared to control plants. In addition, SOD content was significantly increased in the sensitive genotype (Atlantic) compared with the tolerance genotype (Qingshu No. 9). However, the SOD content of the plants treated with thiamethoxam was increased in both genotypes compared to the control and the other treatments. This increase was highest when the thiamethoxam treatment was applied under drought stress, especially in the Atlantic drought-sensitive genotype. This increase in SOD content in the Atlantic could be due to the effect of thiamethoxam. This result shows that thiamethoxam can effectively increase the SOD content in potato, which helps to eliminate superoxide free radicals generated in potato cells under drought stress and improve the resistance of potato.

Yield per plant results show that potato plants treated with thiamethoxam under normal irrigation (T–ND) had the highest yield, followed by the control, while plants under drought stress without the application of thiamethoxam (NT–D) had the lowest yield per plant for both genotypes in 2018. Similar results were obtained with the drought tolerant–genotype Qingshu No. 9 in the 2019 cropping season. However, plants treated with thiamethoxam under drought stress (T–D) had a higher yield per plant than the corresponding control plants in the 2019 growing season. This result indicates that thiamethoxam can effectively increase potato yield under adequate soil moisture and drought stress. In addition, during the two-year harvest period, the average yield per plant of T–D of Qingshu 9 increased by 70.1% compared with NT–D, while that of Atlantic was only 33.01%. The results indicated that thiamethoxam greatly increased the yield of Qingshu No. 9 (drought-tolerant genotype). These results fully indicate that thiamethoxam could effectively improve the drought resistance and yield of potatoes under drought stress, and the effect on drought-tolerant genotype Qingshu No. 9 was better. Principal component analysis of the drought-resistance index of potato under different treatments showed that the total percentage of principal components with characteristic values greater than 1 reached 87.68%. The comprehensive ranking of drought-resistance indices of potato under different treatments was T–D, T–ND, NT–ND and NT–D, and the comprehensive factors and indices were best in treatment T–D.

The production of neonicotinoid pesticides has brought great opportunities to the development of world agriculture, and has now become one of the most widely used insecticides in the world. Their mechanism of action is to cause paralysis or even death of pests by interfering with their nerve conduction. As the second generation of neonicotinoid compounds, thiamethoxam has the characteristics of high efficiency and low toxicity, which not only has obvious advantages in pest control [21] but also can improve crop resistance and yield by promoting crop root growth, tiller number, and flowering, etc., thus making outstanding contributions to agricultural development [23,24,25]. However, everything has two sides. Some studies have reported that thiamethoxam can produce pesticide residues in soil, water, air, agricultural products, etc., posing potential risks to ecosystems, microorganisms, animals and humans [48,49,50,51]. The limitation of this paper is that only the contribution of thiamethoxam to the drought resistance and yield of potatoes has been studied, but the ecological effects of thiamethoxam have not been explored. According to the existing research, the harm of thiamethoxam can be reduced by physical, chemical and microbial means, for example, high pH hydrolysis, high-pressure mercury lamps, and ultraviolet light degradation, and bacterial microbial degradation such as rhizobium can achieve a more satisfactory thiamethoxam degradation effect [52,53,54,55]. Therefore, in future agricultural development, it is imperative to conduct research on plant metabolism and implement supportive agronomic measures. These efforts will provide indispensable theoretical support for the scientific application of thiamethoxam.

## 4. Materials and Methods

### 4.1. Experimental Site and Materials

The experiment was conducted in the College of Agronomy, Gansu Agricultural University Lanzhou, China (36°030′ N; 103°400′ E) (Table 3). Potato genotypes used in this experiment included Qingshu No. 9, which is described as a drought–tolerant genotype, while the Atlantic genotype is considered drought–sensitive [56]. The seed coating agent Ruisheng was purchased from Syngenta and used as a coating agent for seed treatment. The active ingredient thiamethoxam contained in Ruisheng was 70%. Thiamethoxam is used as an insecticide with systemic conductive seed and has a gastric poisoning and contacting effect on insect pests. At the same time, Ruisheng (70% thiamethoxam) can be used as a seed admixture to control the effects of drought on agricultural production.

### 4.2. Experimental Design and Treatments

A 2 × 4 factorial experiment with a completely randomized design (CRD) with twenty-five biological replicates was laid out in a screen house pot experiment. Treatments included the two potato genotypes (Qingshu No. 9 and Atlantic) and four treatment factors. A total of two hundred plastic pots filled with soil were divided into four groups as treatment factors. Half of the treatments, i.e., the first and the third, received normal irrigation without the application of thiamethoxam group. Thus, the first treatment consists of normal irrigation without applying the thiamethoxam group, which is referred to as NT–ND. The second treatment consists of imposition of drought or no irrigation without the application of the thiamethoxam group (NT–D). The third treatment consists of normal irrigation with the application of the thiamethoxam group (T–ND). The last treatment is the imposition of drought and the application of thiamethoxam (T–D). The treatments are summarized in Table 4 below.

The pots used for the experiment have a height of 45 cm and an internal diameter of 43 cm. The medium used is a mixture of topsoil and vermiculite in a ratio of 2:1. The composition of the medium consists of a total nitrogen content of 1.6%, a total phosphorus content of 1.7%, a total potassium content of 1.2%, an organic matter content of 50% and a pH of 7.0 ± 0.2. Each pot was three-quarters filled with the medium. The medium was irrigated until the saturated water content of the soil was reached (θw: 70 ± 5%). The potato seeds were coated with Ruisheng until each seed surface was evenly covered. The seeds were dried at room temperature for 24 h before sowing. Each pot was sown with one potato seed to a depth of about 8 cm in the layer of medium. Each treatment included twenty-five pots with one plant in each. The pot experiment was monitored until harvest time. Morphological and physiological data such as plant height, stem diameter, chlorophyll content and yield were measured. Biochemical data such as MDA, proline, and SOD were measured on the 40th, 60th, 80th, 100th, and 120th days.

### 4.3. Methods of Data Measurements

#### 4.3.1. Determination of Plant Height, Stem Thickness and Yield

Five potato plants were randomly selected from each replicate and the height (cm) was measured with a ruler. The measurement was made from the surface of the soil layer to the first inflorescence of each plant. Stem thickness (mm) was measured with calipers to determine the diameter of the base of the main stem of each plant. These measurements were taken and recorded on plants 40, 60, 80, and 100 days after planting. After the measurements and recordings, the average among five randomly selected plants was determined as a replicate.

After measuring plant height, roots were carefully separated from stems, rinsed in distilled water, and recorded by scanning the roots with Win RHIZO version 5.0 image analysis software (STD) 4800, EPSON, Quebec City, QC, Canada). At the same time, the leaves of the separated plants were immediately immersed in liquid nitrogen and stored in a freezer at −80 °C for biochemical analysis.

#### 4.3.2. Determination of Relative Chlorophyll Content (SPAD Value)

In this study, a portable chlorophyll meter (model SPAD-502, Konica Minolta, Sakai, Osaka, Japan) was used to determine the SPAD value of leaves. Measurements were made on five sampled plants per replicate, and the five recorded values were averaged to obtain a SPAD value for each replicate. The SPAD values were recorded at 40, 60, 80, 100, and 120 days, respectively.

#### 4.3.3. Determination of Malondialdehyde (MDA) and Proline (Pro) Content

Proline content was determined using sulfosalicylic acid, and MDA content was determined using the chromogenic thiobarbituric acid TBA method described by Kumar [57].

#### 4.3.4. Determination of Enzyme Activity of Superoxide Dismutase (SOD)

Superoxide dismutase activity was determined by determining the ability of plants to prevent photochemical reduction of nitroblue tetrazolium (NBT). The amount of reduced NBT was measured by spectrophotometry colorimetry at 560 nm after 50% of NBT photoreduction was inhibited with one unit of SOD activity [17].

#### 4.3.5. Determination of Yield Per Plant

In the potato growth period of 60, 80, 100 and 120 days, 5 plants were randomly selected for sampling and the soil on the potato cubes was rinsed with water, and after the surface moisture dried, the weight of a single plant was weighed and averaged.

### 4.4. Analysis of the Data

SPSS version 22.0 (IBM Corp., Chicago, IL, USA) was used to analyze the collected data. Duncan multiple range tests were used to distinguish the means of the treatments with a probability of 5%. Standard deviations were used to represent the dispersion of the means in the figures. GraphPad Prism version 8.0 (GraphPad Software, Inc., San Diego, CA, USA) and Microsoft Excel 2019 were used to generate all graphs.

## 5. Conclusions

Low soil moisture content, low relative humidity, and high temperatures all contribute to drought stress. If drought persists, plants dry out and production suffers. Drought stress in potatoes results in delayed sprouting, slow plant development, reduced plant mass weight, and a sharp decline in tuber quantity, size, and yield. Thiamethoxam, a substance that effectively controls below-ground pests, can be used to significantly increase the chlorophyll content of leaves during drought to improve resistance. In the present study, the effects of thiamethoxam on two potato genotypes were investigated under four treatments (NT–ND, NT–D, T–ND, T–D) in two years. The results showed that the treatments (T–D and T–ND) with thiamethoxam application performed better under drought stress than treatments without thiamethoxam application (NT–ND and NT–D) in both years. Key antioxidant enzymes, such as SOD, relative chlorophyll content, plant height, stem thickness, and root length increased, Pro content and MDA content decreased, and yield also increased, especially under drought conditions in both genotypes. These significant yield increases were more pronounced 100 and 120 days after sowing in both years. Therefore, thiamethoxam can effectively improve the drought resistance and yield of potato under drought stress, and its effect on the drought-tolerant genotype Qingshu No. 9 is better. A comprehensive evaluation analysis based on principal component analysis was performed. The results evaluated the treatments in this order: T–D > T–ND > NT–ND > NT–D. Th end result showed that the comprehensive factors and indices in potato treatment T–D are the best. The obtained results can be useful for potato growers to reduce the adverse effects of drought on this important crop and maximize yield.

In conclusion, this study demonstrates that thiamethoxam exhibits positive effects on drought resistance and potato yield, but the limitation is that the effects of thiamethoxam on soil microorganisms and whether there are toxic residues in the potatoes were not explored. However, previous studies have shown that the potential negative ecological consequences associated with thiamethoxam can be mitigated through physical, chemical, and biological thiamethoxam degradation methods. Therefore, in potato production, the judicious application of thiamethoxam along with appropriate agronomic practices are strongly recommended to effectively strike a balance between increasing yield and promoting environmental sustainability.

## Figures and Tables

**Table 1:** Principal components and weight values of drought-resistance indices of potatoes in different treatments.

Indictors	PCA 1	PCA 2	Weighted Value
Plant height	0.9547	0.0260	0.1508
Thick stems	0.1984	0.8658	0.0942
Chlorophyll content	0.5656	0.5353	0.1273
Malondialdehyde content	0.8876	0.4211	0.1692
Proline content	0.9755	0.1959	0.1665
Superoxide dismutase content	0.5239	0.8398	0.1431
Yield per plant	0.9461	0.0197	0.1490
Eigen value	4.1796	1.9581	
Contribution (%)	59.7096	27.9734	
Cumulative (%)	59.7096	87.6831	

**Table 2:** Membership degree and comprehensive evaluation index of drought-resistance indices of potato in different treatments.

Indictors	NT–ND	NT–D	T–ND	T–D
Plant height	0.4248	0.0000	1.0000	0.2146
Thick stems	0.6495	0.0000	0.3843	1.0000
Chlorophyll content	0.0000	0.7664	0.5452	1.0000
Malondialdehyde content	0.8624	0.0000	1.0000	0.7248
Proline content	0.3966	1.0000	0.0000	0.5811
Superoxide dismutase content	0.0000	0.3164	0.1258	1.0000
Yield per plant	0.3964	0.0000	1.0000	0.1638
Comprehensive index	0.3962	0.3093	0.5926	0.6407
Comprehensive ranking	3	4	2	1

**Table 3:** Meteorological data of the experimental sites in Gansu Agricultural University from May to September 2018 and 2019.

Year	Month	Average Low Temperature/(°C)	Average High Temperature/(°C)	Average Temperature/(°C)	Average Humidity/(g·kg^−1^)	Rainfall/(mm)
2018	May	10.54	25.15	19.26	34.22	39.3
	June	15.11	28.26	23.35	36.92	11.4
	July	17.69	28.80	23.88	26.79	97.1
	August	18.84	26.45	23.13	23.07	106.4
	September	10.24	21.00	17.18	23.81	93.6
2019	May	10.31	22.12	20.47	20.12	14.5
	June	15.29	26.46	22.35	30.48	66.7
	July	17.27	28.13	23.41	25.11	44.8
	August	17.13	28.49	23.72	27.19	71.6
	September	13.21	24.58	19.46	28.47	52.1

**Table 4:** Experimental design for soil moisture and thiamethoxam treatment.

Treatment Number	Treatment Conditions
NT–ND	Soil volume water content (θw: 55 ± 5%) was not applied to thiamethoxam, throughout the growing season, and there was no drought stress.
NT–D	The soil volume water content (θw: 35 ± 5%) throughout the growing period, without applying thiamethoxam, there was drought stress.
T–ND	The soil volume water content (θw: 55 ± 5%) in the whole growing period was coated with thiamethoxam, and there was no drought stress.
T–D	The soil volume water content (θw: 35 ± 5%) in the whole growing period, coated with thiamethoxam, there was drought stress.

## Data Availability

The entire set of raw data presented in this study is available upon request from the corresponding author.
